# TRAIL Induces Nuclear Translocation and Chromatin Localization of TRAIL Death Receptors

**DOI:** 10.3390/cancers11081167

**Published:** 2019-08-14

**Authors:** Ufuk Mert, Alshaimaa Adawy, Elisabeth Scharff, Pierre Teichmann, Anna Willms, Verena Haselmann, Cynthia Colmorgen, Johannes Lemke, Silvia von Karstedt, Jürgen Fritsch, Anna Trauzold

**Affiliations:** 1 Institute for Experimental Cancer Research, University of Kiel, 24105 Kiel, Germany; 2 Department of Clinical Chemistry, University Medical Centre, Ruprecht-Karls University of Heidelberg, 68167 Mannheim, Germany; 3 Department of General and Visceral Surgery, Ulm University Hospital, Albert-Einstein-Allee 23, 89081 Ulm, Germany; 4 Department of Translational Genomics, Medical Faculty, University of Cologne, 50931 Cologne, Germany; 5 CECAD Research Center, Medical Faculty, University of Cologne, 50931 Cologne, Germany; 6 Department of Infection Prevention and Infectious Diseases, University of Regensburg, 93053 Regensburg, Germany

**Keywords:** TRAIL, nuclear TRAIL-R1, nuclear TRAIL-R2, trafficking, CRM-1

## Abstract

Binding of tumor necrosis factor-related apoptosis-inducing ligand (TRAIL) to the plasma membrane TRAIL-R1/-R2 selectively kills tumor cells. This discovery led to evaluation of TRAIL-R1/-R2 as targets for anti-cancer therapy, yet the corresponding clinical trials were disappointing. Meanwhile, it emerged that many cancer cells are TRAIL-resistant and that TRAIL-R1/-R2-triggering may lead to tumor-promoting effects. Intriguingly, recent studies uncovered specific functions of long ignored intracellular TRAIL-R1/-R2, with tumor-promoting functions of nuclear (n)TRAIL-R2 as the regulator of let-7-maturation. As nuclear trafficking of TRAIL-Rs is not well understood, we addressed this issue in our present study. Cell surface biotinylation and tracking of biotinylated proteins in intracellular compartments revealed that nTRAIL-Rs originate from the plasma membrane. Nuclear TRAIL-Rs-trafficking is a fast process, requiring clathrin-dependent endocytosis and it is TRAIL-dependent. Immunoprecipitation and immunofluorescence approaches revealed an interaction of nTRAIL-R2 with the nucleo-cytoplasmic shuttle protein Exportin-1/CRM-1. Mutation of a putative nuclear export sequence (NES) in TRAIL-R2 or the inhibition of CRM-1 by Leptomycin-B resulted in the nuclear accumulation of TRAIL-R2. In addition, TRAIL-R1 and TRAIL-R2 constitutively localize to chromatin, which is strongly enhanced by TRAIL-treatment. Our data highlight the novel role for surface-activated TRAIL-Rs by direct trafficking and signaling into the nucleus, a previously unknown signaling principle for cell surface receptors that belong to the TNF-superfamily.

## 1. Introduction

Tumor necrosis factor (TNF)-related apoptosis-inducing ligand (TRAIL) binds to four plasma membrane-expressed receptors TRAIL-R1-R4. Two of these receptors, TRAIL-R1/DR4 [1] and TRAIL-R2/DR5/TRICK/Killer [2,3], contain a so-called death domain (DD) and they are capable of inducing apoptosis or necroptosis in response to ligand binding [4,5,6,7]. TRAIL-R3 and TRAIL-R4 do not contain a functional DD and are therefore not able to induce apoptosis [8,9,10]. In contrast, these receptors may inhibit TRAIL-mediated apoptosis, either via direct competition with TRAIL-R1 and TRAIL-R2 for TRAIL binding and/or, as described for TRAIL-R4, via interaction with the death receptors, which results in a non-functional death-inducing signaling complex (DISC) [11,12].

TRAIL gained special attention due to its ability to induce cell death preferentially in tumor cells, while sparing normal cells [13,14]. This led to the development of TRAIL formulations and agonistic anti-TRAIL-R1/-R2 antibodies, which were tested in clinical trials for the treatment of different malignancies (reviewed in [15]). However, initial enthusiasm was dampened by the observations that the majority of primary tumor cells are resistant to TRAIL-treatment. In addition, we and others have shown that, besides inducing cell death, TRAIL death receptors are able to activate several non-apoptotic signaling pathways, such as NF-κB, MAP-kinases, Src, and AKT, which in turn result in proliferation, migration, invasion, and metastasis [16,17,18,19,20,21,22,23,24]. Furthermore, a TRAIL-induced secretome impacts the tumor microenvironment and further enhances the promotion of malignancy [25]. Importantly, the cancer cells are able to produce autocrine TRAIL, which induces migration and tumor metastasis as a specific function of TRAIL-R2 in K-Ras mutated cancers [26].

All the aforementioned functions of TRAIL death receptors are attributed to their presence at the cell surface. Emerging evidence suggests, however, that tumor cells express TRAIL receptors mainly intracellularly, in the cytoplasm and in the nucleus. The high levels of these intracellular receptors, particularly TRAIL-R2, frequently correlate with poor patient’s prognosis (reviewed in [27]). Thus, the intracellular sequestration of TRAIL-R1/-R2 may represent a strategy to escape TRAIL-induced apoptosis [28,29,30,31]. More recently, unexpected specific functions of cytoplasmic and nuclear TRAIL death receptors were uncovered. Cytoplasmic TRAIL-R1 and TRAIL-R2 have been shown to play an essential role during the unfolded protein response (UPR), where they induce apoptosis in the case of an unresolved UPR [32,33]. In contrast, the only known specific function of nuclear TRAIL receptors (nTRAIL-Rs) does not relate to cell death. Instead, nTRAIL-R2 interacts with the microprocessor complex and its accessory proteins, and impairs the maturation of let-7 miRNA via these interactions. This leads to increased levels of the let-7 targets lin28B and HMGA2 and consequently to enhanced cell proliferation [34]. To date, the potential function(s) of nTRAIL-R1 are completely unknown.

Although the presence of TRAIL death receptors in the cytoplasm and the nucleus has been long recognized, the mechanisms of their intracellular, in particular, nuclear trafficking are still poorly explored. The internalization of these receptors by clathrin-dependent endocytosis seems to be a conserved, widespread phenomenon in cancer cells. Thus, treatment with TRAIL induced clathrin-dependent endocytosis of TRAIL-R1 and/or TRAIL-R2 in BJAB Burkitt lymphoma B cells, HeLa human cervical carcinoma, A549 lung cancer, MDA-MB-231 breast cancer, and Huh-7 hepatocellular carcinoma cells [29,35,36,37]. Furthermore, constitutive internalization of both death receptors has been reported for breast cancer cells, an effect that could be blocked by inhibition of clathrin-dependent endocytosis [38]. Whether endogenous, tumor cell-derived TRAIL was a trigger for receptor internalization in these cells is not known. Importantly, whether or not the surface endocytosis of TRAIL receptors precedes nuclear trafficking of TRAIL receptors is equally unknown. Recently, an lmportin-β1-dependent mechanism of TRAIL-R2 nuclear translocation has been proposed [28]. However, the origin of both nuclear TRAIL-R1 and TRAIL-R2, the impact of TRAIL stimulation on their nuclear translocation, as well as shuttling of receptors out of the nucleus remain unanswered. Here, we demonstrate that nuclear TRAIL-Rs directly shuttle from the plasma membrane through initial clathrin-dependent endocytosis in an endogenous and exogenous TRAIL stimulation-dependent manner. Importantly, both of the receptors can be found in chromatin fractions and we identify a novel nuclear export sequence in both TRAIL-Rs. Along these lines, our study highlights a novel signaling principle in TRAIL/TRAIL receptor biology. Given the emerging role of nuclear TRAIL-Rs in promoting tumor progression, gaining knowledge regarding their dynamic trafficking within the cell may reveal novel therapeutic strategies for counteracting nuclear cancer-promoting TRAIL-R activity.

## 2. Results

### 2.1. Plasma Membrane TRAIL-R1 and TRAIL-R2 Translocate to the Nucleus in a TRAIL-Dependent Manner

**Figure 1:**
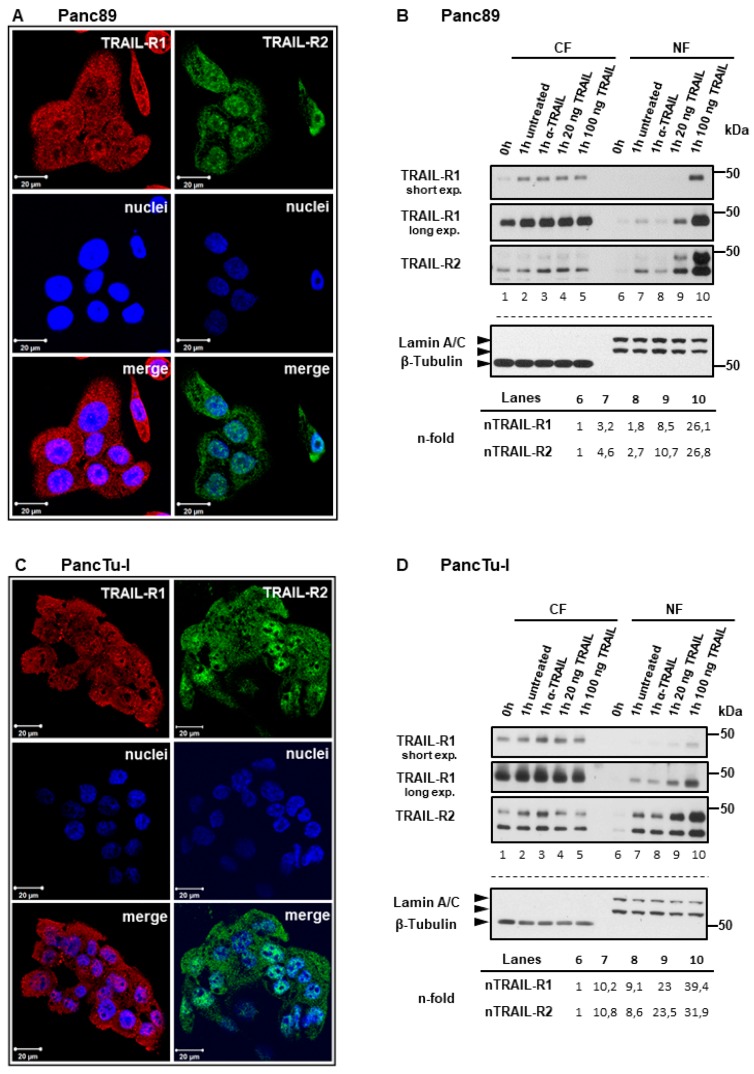
Plasma membrane TRAIL-R1 and TRAIL-R2 translocate to the nucleus in response to tumor necrosis factor-related apoptosis-inducing ligand (TRAIL) stimulation. (**A**,**C**) intracellular distribution of TRAIL receptors analyzed by indirect immunofluorescence and confocal LSM studied in Panc89 and PancTu-I cell lines. Panc89 cells (**B**) and PancTu-I cells (**D**) were surface-labeled with biotin at 4 °C followed by incubation of cells with or without neutralizing anti-TRAIL antibody (α-TRAIL) or indicated concentrations of recombinant TRAIL for one hour at 37 °C. Cytoplasmic fractions (CF) and nuclear fractions (NF) were isolated and biotinylated protein-complexes were affinity purified using streptavidin-conjugated beads. Precipitates were analyzed by Western blotting for the presence of TRAIL-R1 and TRAIL-R2. Protein lysates from both fractions were immunoblotted for Lamin A/C (NF marker) and beta tubulin (CF marker) as control for the equal amounts of extracts used for purification of biotinylated-proteins. Densitometry-based quantifications of TRAIL-R1 and TRAIL-R2 bands are shown below the blots. Band intensities in relation to Lamin C were normalized to corresponding controls.

In cancer cells, TRAIL death receptor expression can be found at the cell surface, in the cytoplasm, and in the nucleus (Figure 1A,C; [27]), yet the origin of nuclear TRAIL receptors is not known so far. To clarify this issue, we biotinylated the plasma membrane proteins, which were then tracked in cytoplasmic and nuclear cell fractions. In detail, we biotinylated PancTu-I and Panc89 cells at 4 °C, transferred them to 37 °C for different time periods, isolated cytoplasmic and nuclear fractions, precipitated the biotin-labeled protein complexes from these samples and analyzed the precipitates for the presence of TRAIL-R1 and TRAIL-R2 by Western blotting. Both death receptors were internalized and stimulation with exogenous TRAIL led to a dramatic increase in nuclear translocation, in addition to the low levels of constitutive nuclear translocation observed (Figure 1B,D, lane 1 vs. 2 and 6 vs. 7, Supplementary Figure S1).

**Figure 2:**
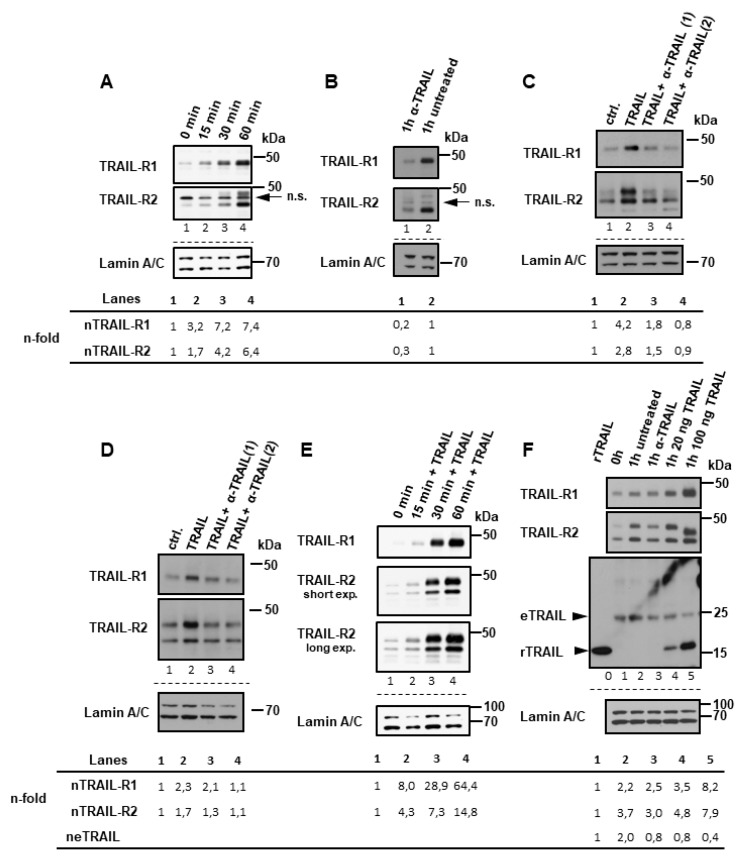
TRAIL receptors translocate to the nucleus in a ligand-dependent manner. Biotin labeling of surface proteins was done at 4 °C in Panc89 cells (**A**–**C**) and PancTu-I (**D**–**F**) cells. Receptor trafficking was restored by incubating cells in full medium at 37 °C for the indicated time points with or without anti-TRAIL neutralizing antibody (α-TRAIL) and with or without different concentration of recombinant TRAIL. Nuclear fractions (NF) were isolated and biotinylated protein-complexes were affinity purified using streptavidin-conjugated beads. Precipitates were analyzed by Western blotting for the presence of TRAIL-R1, TRAIL-R2 (**A**–**F**) or endogenous (e) or recombinant (r)TRAIL (**F**). In lane “0” in (**F**), 35 pg of rTRAIL, which was used for stimulation was loaded. Protein lysates from nuclear fractions were immunoblotted for lamin A/C (NF marker) as control for the equal amounts of extracts used for purification of biotinylated-proteins. (**A**) Panc89 cells were incubated for indicated time periods at 37 °C without any additional stimulation. (**B**) Panc89 cells were incubated with or without α-TRAIL (during biotinylation and subsequent incubation at 37 °C). (**C**) Panc89 cells and PancTu-I cells (**D**) were treated for 1h with TRAIL or TRAIL and α-TRAIL in two different concentrations 1: 0.5 µg/mL; 2: 2.5 µg/mL). (**E**) PancTu-I cells were stimulated with 20 ng/mL of recombinant TRAIL for indicated time periods. (**F**) PancTu-I cells were treated or not with different concentration of recombinant TRAIL or left untreated in presence or absence of α-TRAIL. Densitometry-based quantification of TRAIL-R1, TRAIL-R2 and eTRAIL bands are shown below the blots. Bands intensities in relation to Lamin C were normalized to corresponding controls.

We hypothesized that the internalization of TRAIL-R1/-R2 and their nuclear translocation represents a constitutive and very rapid process since cytoplasmic and nuclear fractions that were free of biotinylated plasma membrane-derived receptors could not be obtained even at very short time points. Consistently, we found clear time-dependent accumulation of both receptors in the nuclei of untreated Panc89 cells over 15–60 min following transfer of the cells from 4 °C to 37 °C (Figure 2A). Of note, incubation of cells with a neutralizing anti-TRAIL antibody (α-TRAIL), in Panc89 cells strongly (Figure 1B lane 8 vs. 7 and Figure 2B) and in PancTuI cells slightly (Figure 1D lane 8 vs. 7 and Figure 2F lane 3 vs. 2), inhibited nuclear translocation of TRAIL-R1 and TRAIL-R2. This suggests an expression of endogenous TRAIL and consequently low level of tonic stimulation of TRAIL receptors. Moreover, the stimulation of cells with exogenous recombinant TRAIL (rTRAIL) dose-dependently enhanced nuclear translocation of both receptors (Figure 1B,D, lanes 9, 10), further supporting a ligand-dependent mechanism. In accordance, TRAIL-induced nuclear translocation of TRAIL-R1 and TRAIL-R2 could be abolished by concomitant exposure of cells to rTRAIL and α-TRAIL (Figure 2C,D). Furthermore, nuclear translocation of TRAIL-R1 and TRAIL-R2 in response to rTRAIL was a rapid and time-dependent process (Figure 2E).

In line with a contributing role for tonic stimulation by endogenous TRAIL (eTRAIL), we also detected a tumor cell-derived TRAIL of approx. 24 kDa in nuclear samples (Figure 2F lanes 1–5), which corresponds to the molecular mass of the cleaved soluble form of TRAIL [39,40]. Since only purified, the biotin-containing protein complexes were analyzed, this TRAIL most likely originated from complexes formed at the plasma membrane. Furthermore, recombinant TRAIL (Figure 2F lanes 4, 5) also readily and dose dependently translocated to the nuclei of TRAIL-treated cells. Interestingly, in samples that were obtained from cells treated with high amounts of recombinant TRAIL, the detected amount of endogenous TRAIL was strongly reduced (Figure 2F lane 5 vs. 2). This argues for competitive binding of rTRAIL and eTRAIL to plasma membrane-expressed TRAIL-R1 and TRAIL-R2.

**Figure 3:**
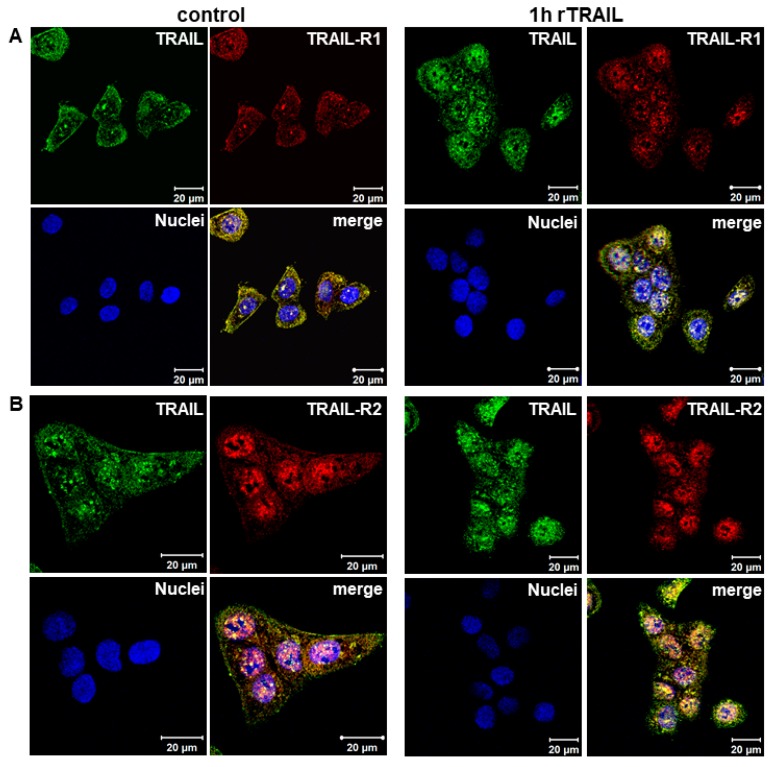
Constitutive and induced nuclear translocation of TRAIL-R1 and TRAIL-R2 (**A**,**B**) Co-localization of TRAIL-R1 and TRAIL-R2 with TRAIL was analyzed by indirect immunofluorescence and confocal LSM in Panc89 cells with or without TRAIL stimulation.

Immunofluorescence studies confirm the presence of endogenous TRAIL in the nucleus as well as its constitutive co-localization with TRAIL-R1 and TRAIL-R2 at the cell surface, in the cytoplasm and, most importantly, also in the nucleus (Figure 3A,B control). Moreover, clearly enhanced nuclear TRAIL staining was detected following the stimulation of cells with rTRAIL, further supporting the data showing the trafficking of rTRAIL to the nucleus. Similarly, enhanced nuclear staining of TRAIL-R1 was observed in response to rTRAIL treatment. A strong increase was not visible for TRAIL-R2, which was probably due to the high levels of constitutive presence of this receptor in the nuclei of untreated cells. Nevertheless, a distinct increase of nuclear co-localisation of both TRAIL-R1 and TRAIL-R2 with TRAIL was detected following treatment with recombinant TRAIL. 

**Figure 4:**
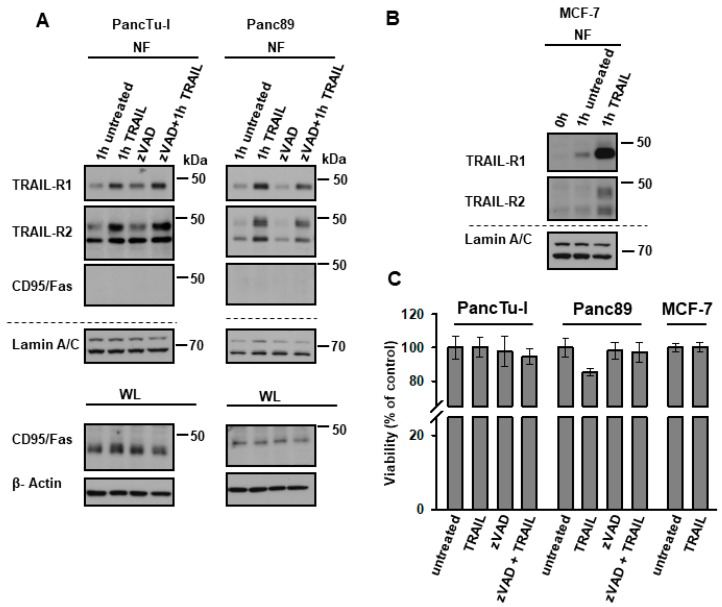
TRAIL-induced nuclear translocation of TRAIL-R1 and TRAIL-R2 is independent of their death-inducing activity. (**A**) PancTu-I cells and Panc89 cells were incubated with or without zVAD-fmk (1 h, 50 µM/mL) followed by biotinylation of surface proteins at 4 °C. Protein trafficking was released for one hour at 37 °C in the presence or absence of recombinant TRAIL (20 ng/mL) and zVAD-fmk. Nuclear fractions (NF) were isolated and biotinylated protein-complexes were affinity purified using streptavidin-conjugated beads. Precipitates were analyzed by Western blotting for the presence of TRAIL-R1, TRAIL-R2 and CD95/Fas. Protein lysates were immunoblotted for Lamin A/C (NF marker) as control for the equal amounts of extracts used for purification of biotinylated-proteins. The corresponding whole lysate (WL) were blotted for CD95/Fas, and β-Actin was used as loading control. (**B**) Biotinylated surface proteins were also tracked in MCF7 cells similar to (**A**). (**C**) Viability assays were performed in PancTu-I, Panc89, and MCF7 cells, as described in Material and Methods. Cells were treated with or without 50 µM/mL zVAD-fmk for 1 h, followed by stimulation with 20 ng/mL recombinant TRAIL for 24 h.

Although PancTu-I and Panc89 are largely resistant to TRAIL [18,41], it is still possible that the nuclear translocation of TRAIL-Rs is just a consequence of the cells dying. To exclude this possibility, we tracked biotinylated receptors in cells that were treated with the pan-caspase inhibitor zVAD-fmk. As shown in Figure 4, TRAIL-R1 and TRAIL-R2 both translocated to the nucleus in response to TRAIL, irrespective of cell death inhibition (Figure 4A). The translocation of TRAIL receptors was specific, since only TRAIL receptors and not CD95/Fas were found enriched in the nucleus following TRAIL-treatment, even though both cell lines express CD95/Fas (Figure 4A). Moreover, TRAIL also induced the translocation of TRAIL-R1 and -R2 in MCF-7 cells (Figure 4B), which are fully resistant to TRAIL-induced cell death (Figure 4C) due to the lack of caspase-3 expression. Therefore, nuclear translocation of TRAIL-Rs is independent of cell death induction.

**Figure 5:**
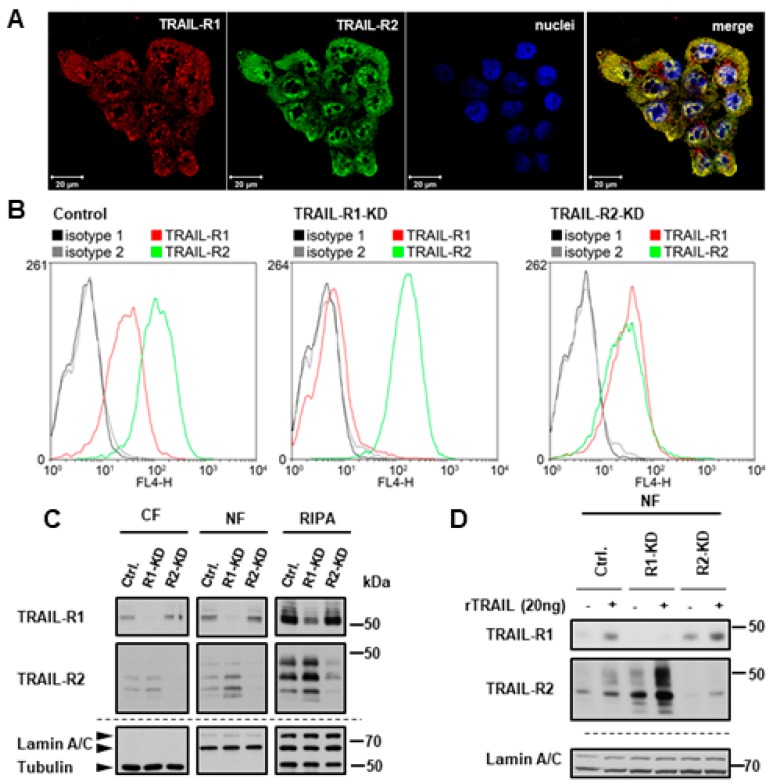
Impact of the interplay of TRAIL death receptors on their nuclear translocation. (**A**) Co-localization of TRAIL-R1 and TRAIL-R2 was analyzed by indirect immunofluorescence and confocal LSM in wild type PancTu-I cells. (**B**–**D**) The expression of TRAIL-R1 and TRAIL-R2 was downregulated in PancTu-I cells by stable transfection with receptor-specific shRNA. (**B**) For analysis of the cell surface levels of TRAIL-R1 and TRAIL-R2 cells were stained with APC-conjugated receptor-specific antibodies and the stainings measured by flow cytometry. Corresponding APC-conjugated isotype controls were used to validate staining specificity. Shown are representative histograms. (**C**) Intracellular distribution of the receptors was studied by Western blot in cytoplasmic fractions (CF), nuclear fractions (NF), and whole cell lysate (RIPA). Detection of β-tubulin (CF marker) and Lamin A/C (NF marker) were used as gel loading control. (**D**) Tracking of biotin-labeled cell surface receptors in the nuclear fractions of cells stimulated for one hour with recombinant TRAIL (rTRAIL) was studied in Western blotting. Protein lysates from nuclear fractions were immunoblotted for Lamin A/C (NF marker) as control for the equal amounts of extracts used for purification of biotinylated-proteins.

We next asked whether nuclear translocation of TRAIL-R1 depends on its interaction with TRAIL-R2 and, vice versa, whether nuclear shuttling of TRAIL-R2 is dependent on the presence of TRAIL-R1. This question is highly relevant, since both death receptors can interact with each other at the plasma membrane with and without TRAIL-treatment. Immunofluorescence staining with confocal LSM-analyses confirmed the mostly intracellular localization of TRAIL-R1 and TRAIL-R2 with TRAIL-R2 being preferentially localized in the nucleus (Figure 5A, Supplementary Figure S2). Interestingly, both receptors co-localized with each other at the plasma membrane, in the cytoplasm and the nucleus.

To clarify the impact of the particular TRAIL receptor on nuclear translocation of the respective other receptor, we generated PancTu-I cells with stable knockdown of TRAIL-R1 or TRAIL-R2. We then tracked the plasma membrane receptors in nuclear fractions of unstimulated and TRAIL-stimulated cells 1 h following their transfer from 4 °C to 37 °C. Remarkably, the knockdown of TRAIL-R1 increased the plasma membrane as well as the overall and nuclear levels of TRAIL-R2 (Figure 5B,C). Consistent with plasma membrane origin of nuclear TRAIL receptors, not only constitutive levels, but also recombinant TRAIL-induced nuclear translocation of TRAIL-R2 was enhanced in TRAIL-R1-KD cells (Figure 5D). Interestingly, the knockdown of TRAIL-R2 did not significantly change either the overall cellular levels of TRAIL-R1 or plasma membrane expression. However, its nuclear translocation was slightly increased (Figure 5D). These data suggest that TRAIL-R1 may function as a negative regulator of TRAIL-induced nuclear trafficking of TRAIL-R2. Importantly, the direct triggering of each receptor via agonistic TRAIL-R1-specific antibody (Mapatumumab; Mapa) or TRAIL-R2-specific antibody (Lexatumumab; Lexa) induced the translocation of the respective receptor alone (Supplementary Figure S3), which points to the principal ability of each receptor to translocate from the plasma membrane to the nucleus following appropriate stimulation.

**Figure 6:**
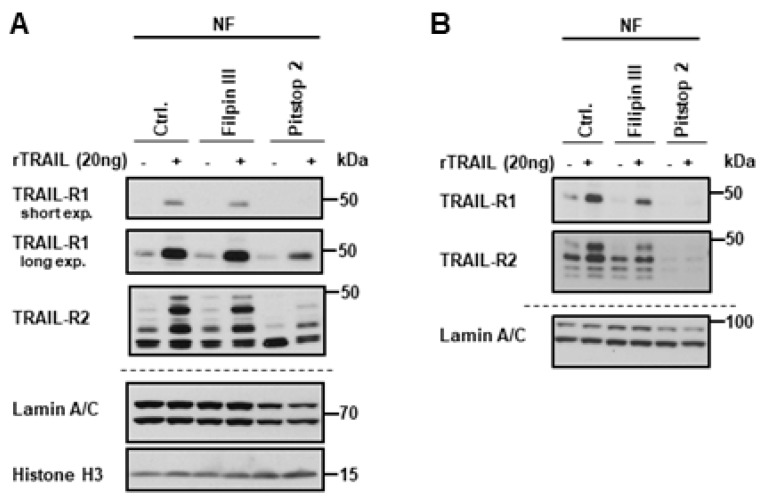
Clathrin-dependent endocytosis is necessary for the nuclear transport of the plasma membrane TRAIL death receptors. Panc89 cells (**A**) and PancTu-I cells (**B**) were incubated with either Filipin III or Pitstop 2. Surface proteins were labeled with biotin and stimulated with 20 ng TRAIL for 1 h. Biotinylated protein complexes were affinity purified from nuclear fractions using streptavidin-coated magnetic beads and analyzed by Western blotting for the presence of TRAIL-R1 and TRAIL-R2. Lysates from nuclear fractions were immunoblotted for nuclear markers (Lamin A/C and Histone H3) as control for the equal amounts of extracts used for purification of biotinylated-proteins.

As we have demonstrated that nTRAIL receptors start their journey at the plasma membrane, we next asked whether receptor-mediated endocytosis is involved in their nuclear translocation. For this purpose, pharmacological inhibitors of either clathrin-dependent endocytosis (CDE; Pitstop 2) or clathrin-independent endocytosis (CIE; Filipin III) were used. Following surface biotinylation with or without TRAIL stimulation, the TRAIL receptors were tracked in the nuclear fraction in the absence or presence of endocytic inhibitors in Panc89 and PancTu-I cells. While total cellular levels of TRAIL-R1 and TRAIL-R2 were not affected by the used inhibitors, the inhibition of CDE dramatically reduced the nuclear levels of both receptors in untreated as well as TRAIL-treated cells (Figure 6A,B). In contrast, only a slight decrease in nuclear levels of TRAIL-R1 and TRAIL-R2 was detected following the blockade of CIE.

Taken together, we found that both TRAIL death receptors translocate constitutively from the plasma membrane to the nucleus and can both in principle do so self-sufficiently, whereas TRAIL-R2 translocation is negatively regulated by TRAIL-R1.

Furthermore, the treatment of cells with recombinant TRAIL dramatically enhances their nuclear translocation time- and dose dependently. Clathrin-mediated endocytosis is necessary for nuclear translocation of both TRAIL-R1 and TRAIL-R2.

### 2.2. Exportin-1/CRM-1 Mediates the Nuclear Export of TRAIL Death Receptors

**Figure 7:**
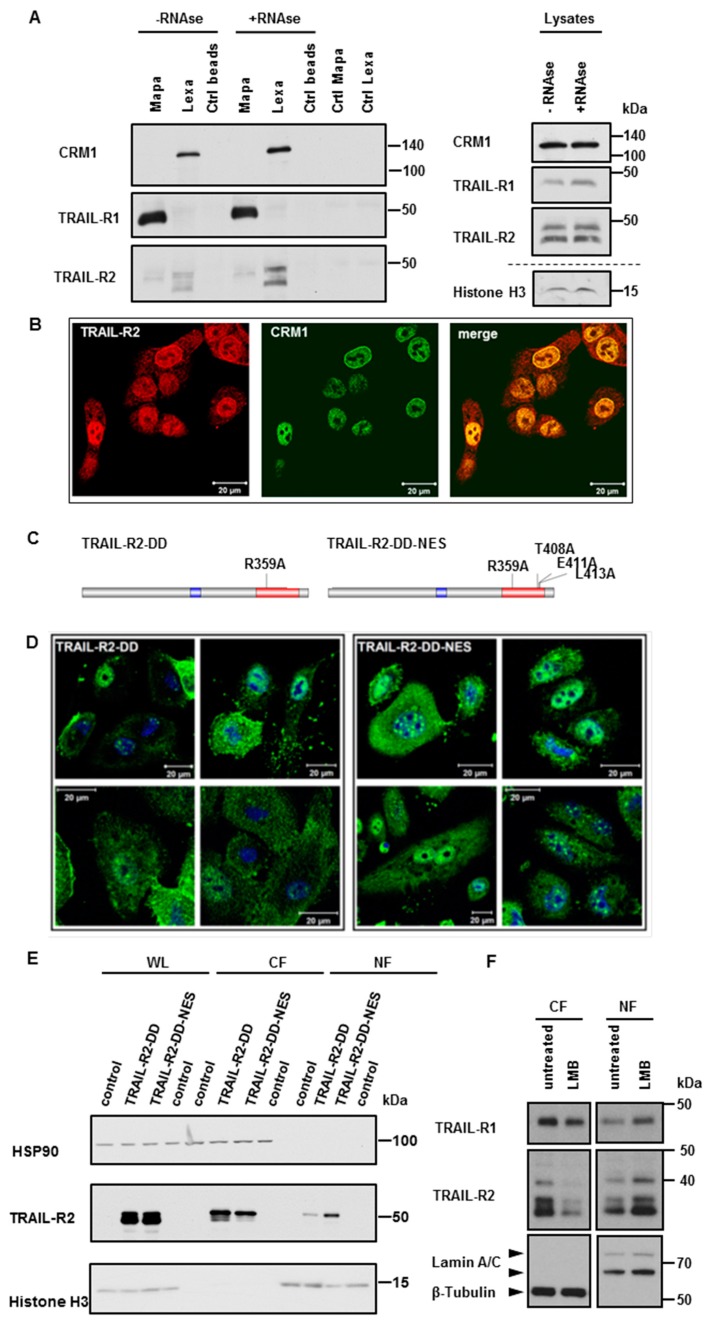
CRM-1 shuttles TRAIL receptors across the nuclear membrane. (**A**) TRAIL-R1 and TRAIL-R2 were immunoprecipitated from nuclear fractions of Panc89 cells using either Mapatumumab (Mapa) or Lexatumumab (Lexa) antibodies, respectively. Protein complexes were analyzed by Western blotting. As control lysates without antibodies (Ctrl beads) and antibodies alone (Ctrl Mapa, Ctrl Lexa) were analyzed in parallel. Immunoprecipitation were also performed with nuclear extracts pre-treated with RNase. Impact of the RNase-treatment on the levels of studied proteins was analyzed by Western blotting. (**B**) Co-localization of TRAIL-R2 with CRM1 in Panc89 cells was analyzed by indirect immunofluorescence and confocal LSM. (**C**) Scheme of TRAIL-R2 constructs with mutated DD (TRAIL-R2-DD) and additional mutations in putative nuclear export sequences (NES; TRAIL-R2-DD-NES). (**D**,**E**) A549 cells with knockout of TRAIL-R2 A549-TRAIL-R2-KO were transiently transfected with expression vectors bearing coding sequence for TRAIL-R2-DD or TRAIL-R2-DD-NES. 24 h later cells were stained by immunofluorescence with antibodies against TRAIL-R2 and analyzed by confocal LSM (**D**) or lyzed and different cell fractions (WL—whole cell lysates; CF—cytoplasmic fraction; NF—nuclear fraction) were analyzed for the expression levels of TRAIL-R2 by Western blotting (**E**). Control cells that were treated with transfection agent alone served as control for TRAIL-R2 overexpression and were analyzed in Western blot in parallel. To control the purity of fractions and an equal gel loading, the expression levels of HSP90 (cytoplasmic fraction) and Histone H3 (nuclear fraction) were analyzed in parallel. (**F**) Panc89 cells were treated with Leptomycin B (LMB) for 8 h. Cytoplasmic fractions (CF), and nuclear fractions (NF) were analyzed by Western blotting for the presence of TRAIL-R1 and TRAIL-R2. Detection of β-Tublin (CF marker) and Lamin A/C (NF marker) served as gel loading control.

While Importin-β1 has been shown to be responsible for nuclear import of TRAIL-R2, the mechanisms of receptor nuclear export are not known. Exportin 1 (XPO 1)/chromosome region maintenance 1-homolog (CRM-1) is the major and best-studied exporter of most nuclear proteins [42]. Analyses of TRAIL-R1/-R2-protein sequences by NetNES 1.1 prediction software [43] predicted the presence of one putative nuclear export sequence (NES) in TRAIL-R1 and three in TRAIL-R2 (Supplementary Figure S4). To test whether CRM-1 could be responsible for nuclear export of TRAIL death receptors, we first tested whether nuclear TRAIL-Rs form complexes with CRM-1. For this purpose, we performed immunoprecipitations of TRAIL-R1 or TRAIL-R2 from nuclear extracts of different PDAC cell lines using receptor-specific antibodies Mapatumumab (anti-TRAIL-R1; Mapa) or Lexatumumab (anti-TRAIL-R2; Lexa) [34,41,44] and analyzed precipitates by Western blot for the presence of CRM-1. Interestingly, we could demonstrate an interaction of CRM-1 with TRAIL-R2, but not with TRAIL-R1 (Figure 7A and Supplementary Figure S5A). Since both, TRAIL-R2 and CRM-1 have been found in complexes with RNA independently of each other [34,42], we also tested the RNA-dependence of the interactions of nTRAIL-R2 with CRM-1. Interactions of TRAIL-R2 with CRM-1 were not abolished, but were even increased upon RNase treatment and can therefore be regarded as protein-protein interactions. Confocal LSM analyses underscored these data, showing the clear and strong co-localization of TRAIL-R2 with CRM-1 in the nucleus and at the nuclear rim (Figure 7B and Supplementary Figure S5B).

To interrogate whether there might be a function for CRM-1-mediated nuclear export of TRAIL-R2, we next constructed two expression vectors encoding for a long isoform of TRAIL-R2 bearing a point mutation in a DD, resulting in a disability of the mutant to bind FADD, and consequently to induce apoptosis (TRAIL-R2-DD) with or without an additional mutation in the putative NES (TRAIL-R2-DD-NES) (Figure 7C). Transient overexpression of these TRAIL-R2 mutants, in A549 cells with stable knock out of TRAIL-R2 revealed clear accumulation of TRAIL-R2 in the nucleus when the CRM-1-binding sequences were mutated (Figure 7E). In line with this finding, immunofluorescence studies confirmed the accumulation of TRAIL-R2-DD-NES in the nuclei of transfected cells (Figure 7D). To further substantiate the role of CRM-1 in nucleocytoplasmic shuttling of TRAIL-R2, we made use of Leptomycin B, a highly specific CRM-1 inhibitor. Leptomycin B binds to the Cys528 residing in the NES-binding groove, thereby preventing cargo binding to CRM1 [45]. Figure 7F demonstrates that the treatment of Panc89 cells with Leptomycin B strongly increased the nuclear levels of TRAIL-R2 while decreasing its cytoplasmic levels in parallel, indicating a critical function for CRM1 activity in regulating nuclear export of TRAIL-R2. Interestingly, less pronounced but similar results were obtained for TRAIL-R1, even though we did not detect an interaction of CRM1 with this receptor by immunoprecipitation, suggesting a weak and less stable interaction with CRM1.

### 2.3. TRAIL-R1 and TRAIL-R2 Translocate into Chromatin-Rich Fractions in a TRAIL-Dependent Manner

Different plasma membrane receptors, like i.e., members of the Epidermal Growth Factor Receptor (EGFR) family, have been shown to translocate into the nucleus in response to stimulation with ligands and have important roles as regulators of DNA repair and replication as well as transcriptional co-activators [46,47]. The latter is accomplished by the physical interaction of these receptors with different transcription factors in the chromatin compartment. So far, the only known specific function of nTRAIL-Rs is the involvement of TRAIL-R2 in the maturation of miRNA let-7. Keeping in mind that nuclear localization of both TRAIL death receptors is a widespread phenomenon it seems likely that these receptors might also have other nuclear functions.

**Figure 8:**
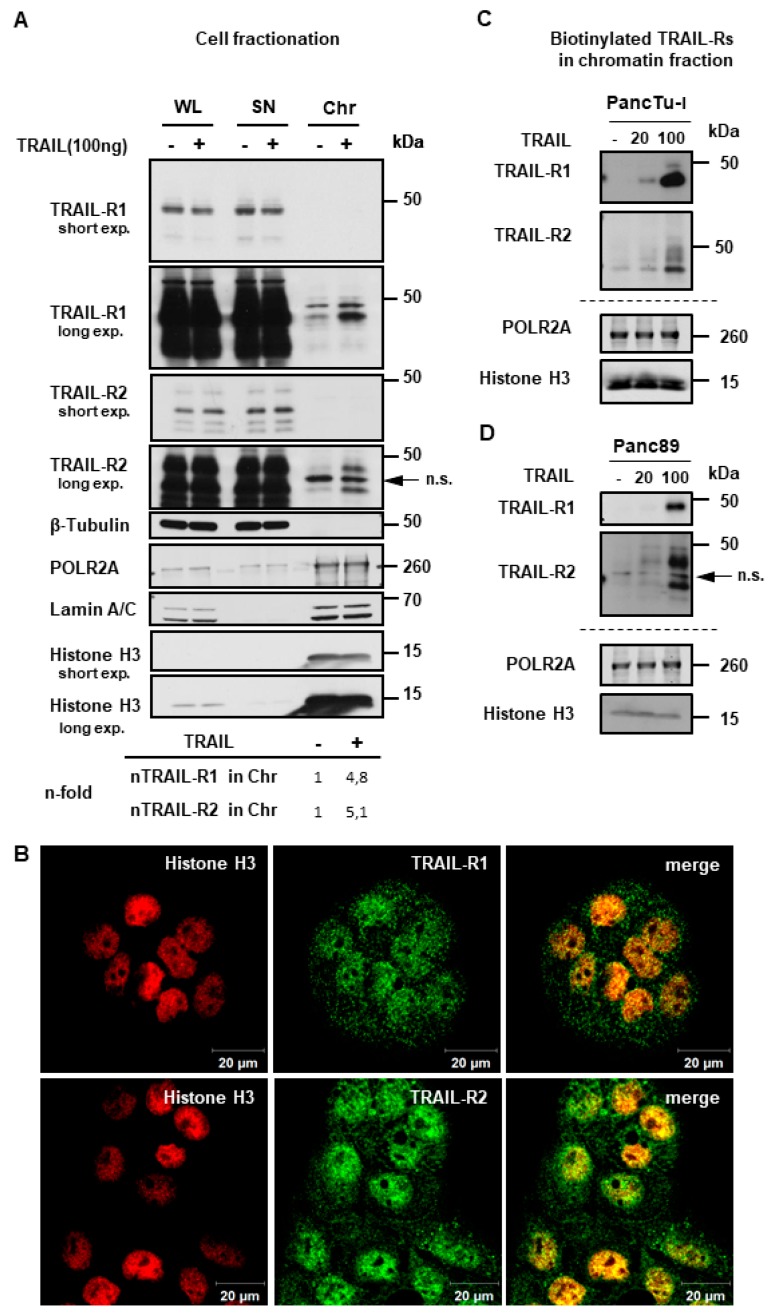
TRAIL-R1 and TRAIL-R2 are enriched in the chromatin fraction upon TRAIL stimulation. (**A**) PancTu-I cells were stimulated with 100 ng TRAIL for one hour. The obtained chromatin fractions (Chr) were analyzed by Western blotting and compared to the supernatant (SN) and to the whole cell lysate (WL). Markers for different subcellular fraction have been included as follows, β-Tubulin (cytoplasmic marker), RNA polymerase 2A (POLR2A) and Histone H3 (chromatin markers), Lamin A/C (nuclear marker). Band intensities of nTRAIL-R1 and nTRAIL-R2 bands were analyzed by densitometry in relation to POL2A and normalized to untreated controls. (**B**) Co-localization of TRAIL-R1 or TRAIL-R2 with Histone H3 in PancTu-I cells was studied by immunofluorescence and confocal LSM. (**C**,**D**) Plasma membrane proteins of PancTu-I and Panc89 cells were labeled with biotin for 1 h at 4 °C, followed by 1 h incubation at 37 °C with or without stimulation with TRAIL in indicated concentrations. Chromatin isolation was done as described in Material and Methods. Biotinylated proteins purified from chromatin fractions were analyzed in Western blot for the presence of TRAIL-R1 and TRAIL-R2. Lysates from chromatin fractions were immunoblotted for nuclear markers (POLR2A and Histone H3) as control for the equal amounts of extracts used for purification of biotinylated-proteins.

To gain more insights into these fascinating novel aspects of TRAIL-R biology, we next tested whether TRAIL-R1 and/or TRAIL-R2 could localize to chromatin. To this end, we purified chromatin fractions from untreated and TRAIL-treated PancTu-I cells and analyzed them by Western blot. The purity of obtained fractions was validated using Histone H3 and RNA polymerase II (as makers of the chromatin fraction) and Tubulin (as a cytoplasmic marker). Indeed, our data revealed the presence of TRAIL-R1 and TRAIL-R2 in the chromatin fraction (Figure 8A). Interestingly, the chromatin-levels of both death receptors were markedly enhanced upon TRAIL-treatment. Samples from whole cell lysates (WL), and supernatants (SN) (representing the cytosol and nucleosol, as described in material and methods), which were included for comparison, showed no changes in the receptors levels in response to TRAIL. Immunofluorescence staining of untreated cells revealed a clear co-localization of both TRAIL-R1 and TRAIL-R2 with Histone H3 (Figure 8B and Supplementary Figure S6). Moreover, the tracking of the biotin-labeled cell surface receptors in response to either 20 ng/mL or 100 ng/mL TRAIL showed a dose-dependent increase of labeled receptors in chromatin fractions of both, PancTu-I and Panc89 cells (Figure 8C,D). Furthermore, the localization of TRAIL death receptors in the chromatin was confirmed in other cells (Supplementary Figure S7), suggesting this to be a common phenomenon in TRAIL-R signaling.

Taken together, our study established surface TRAIL-R1 and -R2 as the source of nTRAIL-Rs, demonstrates that trafficking is independent of caspase activity and it identifies a previously unknown interaction of TRAIL-R2 with CRM-1 and NES. Lastly, we find that nTRAIL-Rs are present in chromatin-rich fractions. Thereby, we provide novel insights into mechanisms of nuclear TRAIL-R trafficking, which we anticipate to contribute to an improved understanding of non-apoptotic TRAIL-R signaling in the future.

## 3. Discussion

Although TRAIL-R1 and TRAIL-R2 are bona fide plasma membrane receptors, they are also present in the cytoplasm and in the nucleus (for review [27]). Particularly, their nuclear localization has long been regarded as a staining artefact and therefore neglected. This attitude hampered the research on possible functions of nuclear TRAIL receptors, which explains why these function(s) remain largely unknown to date. In contrast, the nuclear presence of epidermal growth factor receptor (EGFR) has been noticed already in 1990, and since then cohorts of scientists undertook efforts to understand its nuclear functions. Almost three decades later, a plethora of specific nuclear activities of not only EGFR but also other members of EGFR family and other receptor tyrosine kinases has been deciphered and some crucial aspects of their nuclear translocation pathway have been uncovered (reviewed in [46]).

Concerning TRAIL receptors, to date, only one specific nuclear function has been described [34]. Nuclear TRAIL-R2 regulates let-7 maturation, and thereby promotes the proliferation of different cancer cells in vitro as well as the growth of orthotopically inoculated pancreatic ductal adenocarcinoma cells in vivo in a mouse tumor model. In addition, nuclear sequestration of TRAIL-R2 can act as a resistance mechanism towards TRAIL-mediated apoptosis [28]. Similar to TRAIL-R2, TRAIL-R1 has also been frequently detected in the nucleus [27], but no nuclear functions of TRAIL-R1 have been described so far. Our recent study revealed significantly higher expression of TRAIL-R1 in tumor tissue of pancreatic ductal adenocarcinoma patients (PDAC) as compared to non-neoplastic adjacent tissue [48]. Interestingly, for breast cancer patients, we found a significant association of high nTRAIL-R1 expression with lower tumor stage, less lymphovascular invasion, and reduced rate of local tumor recurrence, suggesting the anti-tumor role of nuclear TRAIL-R1 in this context.

Despite still enigmatic functions of nuclear TRAIL-Rs, the malignancy-enhancing role of nTRAIL-R2 and the obviously widespread phenomenon of nuclear localization of TRAIL death receptors in cancer strongly demands research on the mechanisms of intracellular trafficking of these receptors.

Our present work sheds some light on this aspect of TRAIL-R biology. Here, we demonstrated that both nuclear TRAIL death receptors originate from the plasma membrane. They translocate to the nucleus in response to ligand binding in a process that requires clathrin-dependent endocytosis. Interestingly, in addition to plasma membrane-derived TRAIL-R1 and TRAIL-R2, we also detected endogenous as well as recombinant TRAIL in the nucleus, and both of them showed nuclear co-localization with TRAIL-Rs. Since these ligands were present in the biotinylated fraction, they most likely originate from the plasma membrane and then translocate to the nucleus bound to TRAIL-R1 and/or TRAIL-R2. Interestingly, stimulation with recombinant TRAIL clearly diminished the amount of endogenous TRAIL in the nucleus. This suggests that recombinant TRAIL competes with the endogenous ligand for receptor binding. Of Note, TRAIL induced TRAIL-Rs nuclear localization independent of its cell death-inducing activity. Importantly, TRAIL-R1 limited nuclear trafficking of TRAIL-R2, thereby potentially restricting its tumor-promoting nuclear activity.

Interestingly, together with their cognate full-length or cleaved plasma membrane receptors, different ligands, i.e., EGF, FGF, or NGF, can also translocate into the nucleus (reviewed in [49]), suggesting common mechanisms underlying the stimulation-dependent nuclear translocation of plasma membrane receptors.

One of the best understood pathway by which plasma membrane receptors are moved to the nucleus is that used by EGFR, a prototype member of the EGFR family (reviewed in [46]). Following ligand binding at the cell surface, this receptor is internalized via clathrin-dependent endocytosis, being moved by retrograde vesicular transport towards the endoplasmic reticulum (ER) and subsequently translocate through the ER membrane into the cytosol by action of the Sec61 translocon [50]. Free cytosolic full-length receptors can then be bound at their nuclear localization motif (NLS) by Importin-β and finally imported into the nucleus through the nuclear pore complex [51]. Of note, the Importin-β-mediated nuclear transport of TRAIL-R2 has been recently proposed [28]. However, further investigations are needed to bridge the gap between endocytic vesicles and Importin-β-dependent nuclear entry.

To the best of our knowledge, the mechanisms of nuclear export of TRAIL-R2 were not known. Our present data suggest the involvement of CRM-1, which is an essential cellular transport protein, shuttles a broad range of cargo (proteins, RNA) between nucleus and cytoplasm [42], in TRAIL-R2 nuclear export. While using immunoprecipitation, we detected a strong interaction of CRM-1 with TRAIL-R2, but not with TRAIL-R1. In addition, immunofluorescence staining and confocal LSM analyses demonstrated the interaction of TRAIL-R2 with CRM-1 in the nucleus and most interestingly at the nuclear rim. Mutation of the potential NES predicted in TRAIL-R2 resulted in its nuclear accumulation. Furthermore, the inhibition of nuclear export by Leptomycin B, a highly specific CRM-1-inhibitor, strongly increased the levels of TRAIL-R2 in the nucleus and in parallel decreased its levels in the cytoplasm. Although Leptomycin B also changed the nuclear-cytoplasmatic distribution of TRAIL-R1, we could not detect interaction of TRAIL-R1 with CRM-1 by using immunoprecipitation in any of the cells studied, suggesting either a weak and potentially less stable TRAIL-R1-CRM1 interaction, or a possible role of TRAIL-R2 in shuttling TRAIL-R1 out of the nucleus, which requires further studies. The affinities of NES-containing cargos for their cognate receptor CRM1 can dramatically vary, with low-affinity cargos prevailing [52,53]. Weak affinities seem to be important for efficient disassembly of export complexes on the cytoplasmic side of the nuclear pore complex [54]. Furthermore, they prevent cargos from binding to CRM1 in the cytoplasm in the absence of RanGTP [55].

Regarding the possible nuclear functions of TRAIL death receptors, our present study demonstrates the presence of biotin-labeled TRAIL-R1 and TRAIL-R2 in the chromatin fraction. Furthermore, we found that TRAIL-treatment strongly increased the chromatin-localization of both receptors in a dose-dependent manner. In addition, immunofluorescence staining of both receptors showed co-localization with the chromatin marker Histone H3.

Chromatin-related nuclear functions of different members of Receptor Tyrosin Kinases (RTKs) have already been well documented [46,56]. Nuclear EGFRs, for instance, interact with classical transcription factors e.g., STAT3, STAT5, E2F1, and activate the transcription of respective target genes, such as iNOS, COX-2, Aurora-A, B-Myb [57,58,59,60]. Nuclear EGFR1, in addition, cooperates with RNA helicase A (RHA) to trans-activate the cyclin-D1 gene [61]. Importantly, besides RTKs, other types of cell surface transmembrane receptors are also found in the nucleus, where they exert regulatory functions in gene expression. One such example provides the type I transforming growth factor beta receptor (TβR1), which can localize to the nucleus following TGF-β treatment [62]. In ErbB-2 transformed MDA-MB-231 breast cancer cells nuclear TβR1 interacts with hnRNPA1, which serves as a cofactor for the association with purine-rich RNA sequences [63]. In addition, TβR1-hnRNPA1 complexes stimulate the alternative splicing of pre-mRNA for EGFR1, resulting in the generation of a truncated soluble EGFR isoform. Of note, we have recently shown a strong and direct interaction of hnRNP-A1 with nTRAIL-R2 and TRAIL-R1, although the latter appeared to be less pronounced [34]. CD40 and the B-cell activating factor receptor (BAFF-R), both cytokine receptors and members of the TNF receptor superfamily, also localize to the nucleus in normal and malignant B cells, in addition to their plasma membrane expression, where they interact with c-Rel and regulate gene transcription [64,65].

The interaction of TRAIL-R1 and TRAIL-R2 with chromatin suggests that these receptors can also be involved in the regulation of gene expression and/or DNA metabolism. However, this remains to be elucidated in future studies.

Last but not least, it is also conceivable that TRAIL death receptors in the nucleus exert functions that are related to their canonical functions as inducer of cell death and/or non-apoptotic signaling. Importantly, proteins that are crucially involved in these signaling pathways i.e., FADD, caspase-8, and RIPK1, are also present in the nucleus, thus making such a scenario feasible [66,67,68,69,70,71]. Nevertheless, nuclear translocation was independent of caspase activity, and therefore suggests fulfilling caspase-activity-independent functions. In this regard, it is tempting to speculate whether TRAIL-R4, which only expresses a truncated DD, and therefore cannot activate caspases, might also play a yet unidentified role in nuclear functions of TRAIL-Rs.

Similar observations have been made for EGFRs. Besides interaction with transcription factors, nuclear EGFRs can also “make use of their canonical activities” and phosphorylate nuclear proteins, like PCNA [72] and DNA-dependent protein kinase (DNA-PK) [47,73,74], thereby influencing a DNA replication and DNA damage repair. 

More than 20 years ago, the exciting discovery that TRAIL bears the potential to selectively kill tumor cell upon systemic administration has raised great attention to exploit TRAIL-signaling for cancer treatment. Despite the tremendous efforts to develop TRAIL-Receptor agonists for clinical application, this strategy failed so far, which was most likely due to intrinsic apoptosis resistance of most primary cancer cells (reviewed in [75]). Within the last decade, several groups focused their research on developing TRAIL-sensitizing strategies to eventually circumvent TRAIL-resistance and bring TRAIL-based therapies back into the clinics. Nevertheless, in recent years, much evidence has accumulated that TRAIL-Receptors, besides inducing apoptosis, frequently stimulate non-cell death pathways thereby promoting the malignant features of cancer cell such as proliferation, invasion and migration. Moreover, it has been demonstrated that the TRAIL-Receptors are able to modulate the tumor microenvironment in a pro-tumorigenic manner by stimulating cytokine secretion in tumor cells. Paradoxically, based on this recently gained knowledge, strategies to rather block TRAIL-R’s activity than stimulating it are emerging in cancer therapy. Furthermore, for many years, it was widely neglected that TRAIL-receptors are not only expressed on the plasma membrane, but in large amounts also in other cellular compartments, most importantly the cytoplasm and the nucleus. These findings underline the idea that the TRAIL-Receptors of other cellular compartments than the plasma membrane may play important roles in various signaling pathways, of which—most likely—the majority are still uncovered. Thus, when interfering with the TRAIL-signaling, pro-inflammatory signaling, let-7-related functions, as well as still unknown nuclear/chromatin-related functions of TRAIL-Rs, and not only their death-inducing capacities, must be taken into account. Consequently, a deeper understanding of these recently discovered novel aspects of TRAIL-signaling is urgently warranted. 

In this respect, it will be especially important to identify further nuclear functions of TRAIL-Rs and to characterize the mechanisms of their shuttling between different cell compartments in detail. This may open new opportunities for targeted therapeutic interventions in the future.

## 4. Materials and Methods

### 4.1. Cell Culture

The human pancreatic cancer cell lines PancTu-I, Panc89, and Colo357, breast cancer cells MDA-MB-231, and MCF-7, and lung cancer cells A549 with the knockout of TRAIL-R2 (TRAIL-R2-KO, kindly provided by H. Walczak) were cultured in RPMI 1640 media supplemented with 10% FCS, 1 mM GlutaMAX, and 1 mM sodium pyruvate. The cells were grown for 36 h before running experiments. For stable knockdown of TRAIL-R1 or TRAIL-R2, cells were transduced with the GIPZ Lentiviral Human TNFRSF10A shRNA (cloneID: V3LHS_383714) or GIPZ Lentiviral Human TNFRSF10B shRNA (cloneID: V2LHS_16711), respectively, and selected with puromycin (1 µg/mL). The control cells were transduced with non-silencing control vector. All vectors were purchased from Dharmacon, GE Healthcare, Lafayette, CO, USA. To inhibit nucleo-cytoplasmic translocation, the cells were treated either with 20 nM Leptomycin B (Sigma-Aldrich, Taufkirchen, Germany) or solvent for 8 h, followed by fractionation, as described below.

### 4.2. Nuclear Fractionation

Subcellular fractionation was done, as shown previously, with some modifications [76]. Briefly, 7 × 10^6^ cells were seeded 36 h before the experiment. The cells were lysed in hypotonic buffer (40 mM HEPES, 3 mM MgCl_2_, 20 mM KCl, 2 mM EDTA) supplemented with Complete Protease Inhibitor Cocktail and PhosSTOP (both from Roche, Mannheim, Germany), followed by homogenization while using a syringe with 26-gauge needle. After 15 min incubation and occasionally vortexing at 4 °C, the nuclei were pelleted by centrifugation at 1400× *g* for 10 min at 4 °C, the supernatant was further centrifuged at maximum speed, and the new supernatant was kept as a cytoplasmic fraction. The nuclear pellets were resuspended and incubated for 30 min at 4 °C with hypotonic buffer containing 1% IGEPAL, followed by pelleting by centrifugation at 7000× *g* for 10 min. Washing of nuclear pellets were done 1× with hypotonic buffer and 2× with isotonic sucrose buffer (250 mM Sucrose, 6 mM MgCl_2_, 10 mM Tris-HCl, 0.5% Triton X-100) with centrifugation steps in between at 1400× *g* for 10 min at 4 °C. Finally. nuclear membranes were lysed with 300 µL high salt buffer (40 mM HEPES, 3 mM MgCl_2_, 20 mM KCl, 2 mM EDTA, 500 mM KCl, 20% Glycerol) supplemented with 125 units Benzonase (EMD Millipore, Billerica, MA, USA), protease, and phosphatase inhibitors, followed by 30 min incubation on the wheel at 4 °C. The nuclear fractions were cleared by a final centrifugation step at maximum speed for 10 min.

### 4.3. Chromatin Fractionation

Chromatin fractionation was done, as mentioned previously, with some modifications [77]. Briefly, the cells were stimulated with or without 20 ng/mL or 100 ng/mL rTRAIL (PeproTech, Hamburg, Germany) for 1 h. Cells were collected in ice-cold chromatin purification buffer (10 mM HEPES, pH 7.5, 100 mM NaCl, 3 mM MgCl_2_, 1 mM EGTA, 300 mM sucrose, 0.5% Triton X-100) supplemented with protease and phosphatase inhibitors and incubated on ice for 15 min to permeabilize cells. After centrifugation at 4 °C for 3 min at 5000× *g*, the supernatant (SN) representing the cytosol and nucleosol was collected. The pellet was washed once with 500 µL of the same buffer and collected again by centrifugation. DNA was then digested with 200 U/mL Benzonase in 200 µL chromatin purification buffer for 30 min at room temperature. The pellet was collected again by centrifugation and the supernatant was discarded. After a single wash of the pellet with 500 µL of the ice-cold buffer, the residual chromatin was extracted with 200 µL cold buffer containing 0.25 M ammonium sulfate for 5 min at room temperature. Final centrifugation was carried out at 4 °C for 3 min at 5000× *g* and the supernatant was kept as chromatin fraction (Chr). For the tracking of surface TRAIL-Rs in the chromatin fraction, cell surface protein were biotin labeled, as described later, followed by TRAIL stimulation and the isolation of chromatin fractions.

### 4.4. Cell Surface Protein Labeling

7 × 10^6^ cells were seeded on 150 mm plates and then cultured for 36 h. After removal of the medium, cells were washed twice with ice-cold PBS and incubated with 10 mL.

Sulfo-NHS-SS-Biotin (0.24 mg/mL in PBS) (Thermo Fisher Scientific, Waltham, MA, USA) while being gently shaken on ice for 15 min. Then, the cells were washed with ice cold PBS once and incubated for 10 min with 10 mL quenching solution (0.1 mM CaCl_2_, 1 mM MgCl_2_, 100 mM glycine). After two times wash with ice-cold PBS, trafficking of membrane-bound receptors was resumed by incubating the cells at 37 °C with medium containing either 0.5 µg/mL anti-TRAIL (R&D Systems, Minneapolis, MN, USA) 20 ng/mL or 100 ng/mL rTRAIL for 1 h. Subcellular fractionation was done, as mentioned above, followed by affinity purification of the biotin-labeled proteins while using streptavidin magnetic beads (Thermo Fisher Scientific, Waltham, MA, USA). Biotin-labeled proteins/beads complex were allowed to form overnight, followed by two times wash with TBS containing 0.1% Tween 20. Elution of the protein was achieved by heating the beads at 95 °C for 7 min in reducing the sample buffer and analyzed by immunoblotting.

### 4.5. Flow Cytometric Analyses of Cell Surface Expression of TRAIL Receptors

Cell surface expression levels of TRAIL receptors were analyzed by flow cytometry. Briefly, the cells were detached from culture dishes by treatment with Accutase (Merck, Millipore, Darmstadt, Germany). Afterwards, the cells were washed with cold wash buffer (PBS supplemented with 0.5% BSA and 0.05% sodium azide) and FcR-blocking was performed with human FcR blocking reagent (Miltenyi Biotec GmbH, Bergisch-Galdbach, Germany), according to the manufacturer’s instructions. For single staining of TRAIL receptors, 2 × 10^5^ cells were incubated for 30 min at 4 °C with the following APC-conjugated antibodies: anti-human TRAIL-R1 (clone #69036; 10 µg/mL) or anti-human TRAIL-R2 (clone #71908; 10 µg/mL), which were both purchased from R&D Systems GmbH, Wiesbaden, Germany. Respective isotype control stainings were performed with APC-conjugated mouse IgG_1_ (clone #11711) and mouse IgG_2B_ (clone #13303) antibodies (both from R&D Systems GmbH). Finally, the cells were washed twice in cold wash buffer, resuspended in cold wash buffer supplemented with 1% PFA, and measured within 24 h while using a FACSCalibur (Becton Dickinson, Heidelberg, Germany). A population size of 10,000 cells was regarded as representative for data evaluation using WEASEL v3.0.1 (WEHI, Melbourne, Australia).

### 4.6. TRAIL Receptors Internalization Assay

Clathrin-dependent endocytosis and clathrin-independent endocytosis were blocked by incubating the cells with either 25 µM Pitstop2 (Abcam, Berlin, Germany) for 30 min or 1 µg/mL Filipin III (Sigma-Aldrich, Munich, Germany) for 1 h, respectively. Labeling of cell surface protein was performed, as described above, followed by subcellular fractionation and affinity purification of the labeled-proteins with streptavidin magnetic beads. The eluted proteins were analyzed by immunoblotting.

### 4.7. Immunofluorescence Staining

1 × 10^5^ cells were seeded on glass cover slip and allowed to grow for 36 h. The cells were fixed and permeabilized with 2.5% PFA in TBS for 10 min on ice, followed by incubation with ice cold (−20 °C) methanol for another 10 min separated by 2× wash with ice-cold PBS. After blocking with 1% BSA in TBS, the cells were incubated with primary antibodies diluted in 1% BSA/TBS overnight at 4 °C. Primary antibodies used include: anti-TRAIL-R1 HS101 (Adipogen, Liestal, Switzerland) and Mapatumumab (Human Genome Sciences, Rockville, MD, USA), anti-TRAIL-R2 HS201 (Adipogen) and HGS2 (Human Genome Sciences), anti-Histone H3 [(9715); Cell Signaling, Frankfurt, Germany]. Cells were washed 3× with TBS, followed by incubation with secondary Alexa Fluor labelled antibody (Invitrogen, Darmstadt, Germany) and Hoechst 33,258 in the dark for 1 h at room temperature. The cells were washed 2× with TBS and 1× with aqua distilled water before mounting on glass slide while using Immunoselect Antifading Mounting Medium (Dianova, Hamburg, Germany). Confocal LSM analysis was performed with a Zeiss LSM 510 (Carl Zeiss Jena, Germany). All of the secondary antibodies (Alexa Fluor 546 goat anti-human IgG, Alexa Fluor 488 goat anti-mouse IgG) were obtained from Molecular Probes (Invitrogen).

### 4.8. Western Blot Analysis

Cells lysates were supplemented with Complete Protease Inhibitor Cocktail and PhosphoStop (both from Roche, Mannheim, Germany). Western blot analyses were performed, as described previously [18]. Antibodies used for western blots were purchased from: Cell Signaling, Frankfurt, Germany (anti-TRAIL-R2 (3696), anti-Lamin A/C (2032), anti-Histone H3 (9715)), Epitomics, Burlingame CA, USA (anti-beta Tubulin (1879), anti-alpha Tubulin (1878)), Merck Millipore, Darmstadt, Germany (anti-TRAIL-R1 (AB16955)), ProScience Incorporated, Poway CA, USA (anti-TRAIL-R2 (2019)), Sigma-Aldrich, Munich, Germany (anti-TRAIL (T9191), anti-β-actin (A5441)), BD Bioscience, Heidelberg, Germany (anti-CRM-1 (611833)), Thermo Scientific, Waltham, USA, (anti-POLR2A (MA1-46093)).

### 4.9. Immunoprecipitation

The cells were lyzed in hypotonic lysis buffer (HLB: 10 mM HEPES pH 7.0, 10 mM KCl, 0.2 mM EDTA, 1 mM DTT, Complete^®^ (Roche Diagnostics, Mannheim, Germany) and then centrifuged (6 min, 14,000× *g*, 4 °C). Pellets were washed 2× with HLB, and resuspended in buffer containing 30 mM Tris-HCl, pH 7.5, 120 mM NaCl and 1% glycerol. After homogenization and centrifugation, nuclear extracts were adjusted with NP-40 to a concentration of 0.5% and supplemented with either RNasin (Promega, Manheim, Germany) or RNase A (Sigma-Aldrich, Munich, Germany) and incubated for 1 h at 37 °C to prevent or to perform RNA degradation, respectively. Immunoprecipitations from nuclear extracts (1.5 mg/IP) were done, as described in [17].

### 4.10. Generation and Expression of TRAIL-R2 Constructs

The contructs were synthesized and directly cloned into pCDNA3.1 expression plasmid by Geneart (Regensburg, Germany) based on the Uniprot entry (O14763, TR10B_HUMAN). The following mutations were inserted into the wt sequence: R359A to prevent FADD interaction [78]. Additionally, the predicted NES was mutated by changing: T408A, E411A, L413A. For expression, the plasmids were transfected into A549 TRAIL-R2-KO cells while using reverse transfection and lipofectamin 2000, according to the manufacturer’s instructions. Expression and intracellular localization of TRAIL-R2 were analyzed in cellular fractions and in fixed cells by Western blotting and immunofluorescence with confocal LSM, respectively.

## 5. Conclusions

Our study revealed that both tumor cell derived and recombinant TRAIL-independent of their cell death inducing activity-trigger trafficking of surface TRAIL-R1 and TRAIL-R2 to the nucleus. Clathrin dependent endocytosis was found to be crucial for nuclear translocation of TRAIL receptors, while CRM1 assured their transient presence in the nucleus by shuttling them into the cytoplasm. Nuclear TRAIL-R1/-R2 interact with chromatin, which suggests their role in transcriptional regulation and/or DNA metabolism. In conclusion, our data provide insights into the molecular mechanisms of nuclear trafficking of TRAIL death receptors, which could lead to design of novel therapeutic strategies to counteract nuclear TRAIL-R’s activity in cancer. In addition, our findings concerning interaction of TRAIL-Rs with chromatin open new perspectives on the functions of long neglected intracellular TRAIL-Rs.

## Supplementary Materials

The following are available online at https://www.mdpi.com/2072-6694/11/8/1167/s1, Figure S1: Plasma membrane TRAIL-R1 and TRAIL-R2 translocate to the nucleus in MDA-MB-231, Figure S2: Subcellular co-localization of TRAIL-R1 and TRAIL-R2 in Panc89 cells, Figure S3: Treatment with agonistic TRAIL-R-specific antibodies induce nuclear translocation of the respective receptor, Figure S4: Predicted nuclear export sequence (NES) in TRAIL-R1 and TRAIL-R2 analyzed by NetNES 1.1 prediction software, Figure S5: TRAIL-R2 interacts with CRM-1 in Panc-1 cells, Figure S6: Co-localization of TRAIL-R1 or TRAIL-R2 with Histone H3 in Panc89 cells studied by immunofluorescence and confocal LSM, Figure S7: TRAIL death receptors localized in the chromatin, Chromatin fractions (Chr) and supernatants (SN) from breast cancer cell lines (MDA-MB-231 and MCF7) and pancreatic cancer cell line (Colo357) were analyzed by immunoblotting using the indicated antibody. Subcellular fractionation was done as mentioned previously [79].
