# Supplementary material for: TRAIL Induces Nuclear Translocation and Chromatin Localization of TRAIL Death Receptors
Source: Cancers (Basel). 2019 Aug 14;11(8):1167. doi: 10.3390/cancers11081167 (PMC6721811; doi:10.3390/cancers11081167)
Supplement: Supplementary file 1 [file cancers-11-01167-s001.zip › cancers-576306-SI.pdf]

Supplementary Materials:

TRAIL Induces Nuclear Translocation and Chromatin Localization of TRAIL Death Receptors

Ufuk Mert, Alshaimaa Adawy, Elisabeth Scharff, Pierre Teichmann, Anna Willms, Verena Haselmann, Cynthia Colmorgen, Johannes Lemke, Silvia von Karstedt, Jürgen Fritsch, and Anna Trauzold

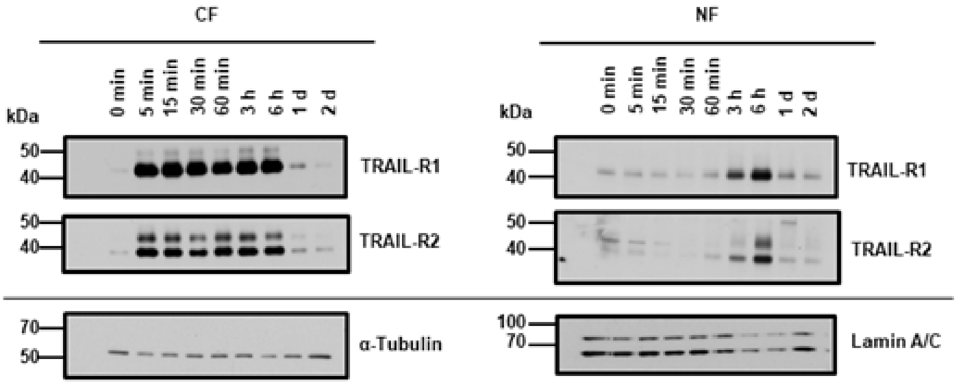

Figure S1. Plasma membrane TRAIL-R1 and TRAIL-R2 translocate to the nucleus in MDA-MB-231. Biotin labeled cell surface receptors were tracked in the cytoplasmic (CF) and nuclear (NF) fractions of MDA-MB-231 cells for the indicated time points. Affinity purification of the labeled proteins was performed using Streptavidin beads followed by analysis in Western blotting. Lysates from the corresponding fractions were tested for Lamin A/C (NF marker) and beta tubulin (CF marker) as control for the equal amounts of extracts used for purification of biotinylated-proteins.

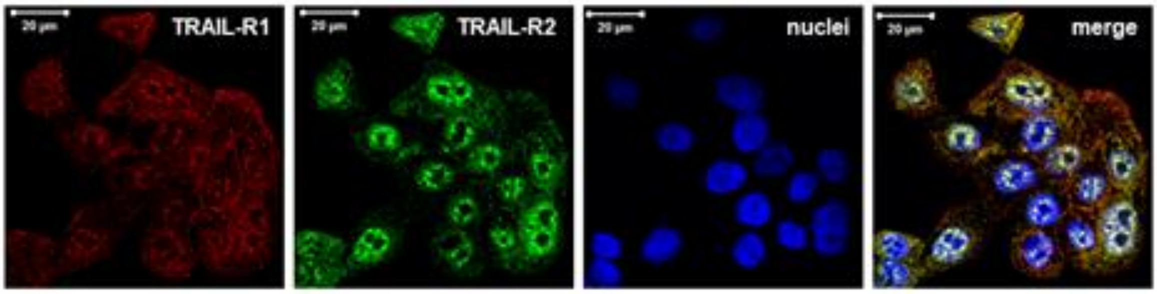

Figure S2. Subcellular co-localization of TRAIL-R1 and TRAIL-R2 in Panc89 cells. Labeling of TRAIL-R1 and TRAIL-R2 in Panc89 cells was done by indirect immunofluorescence and analyzed by confocal LSM.

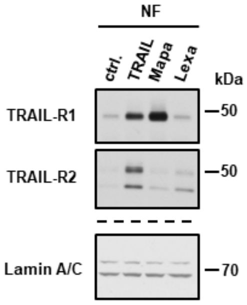

Figure S3. Treatment with agonistic TRAIL-R-specific antibodies induce nuclear translocation of the respective receptor. Surface proteins of PancTu-I cells were labeled with biotin at 4 °C followed by

incubation at 37 °C in medium supplemented or not with TRAIL (20 ng/mL), Mapatumumab (Mapa; 5 μg/mL) or Lexatumumab (Lexa; 5 μg/mL) for 1 h. Biotinylated protein complexes were affinity purified from nuclear fractions using streptavidin-coated magnetic beads and analyzed by Western blotting for the presence of TRAIL-R1 and TRAIL-R2. Nuclear lysates were immunoblotted for nuclear marker (Lamin A/C) as control for the equal amounts of extracts used for purification of biotinylated-proteins.

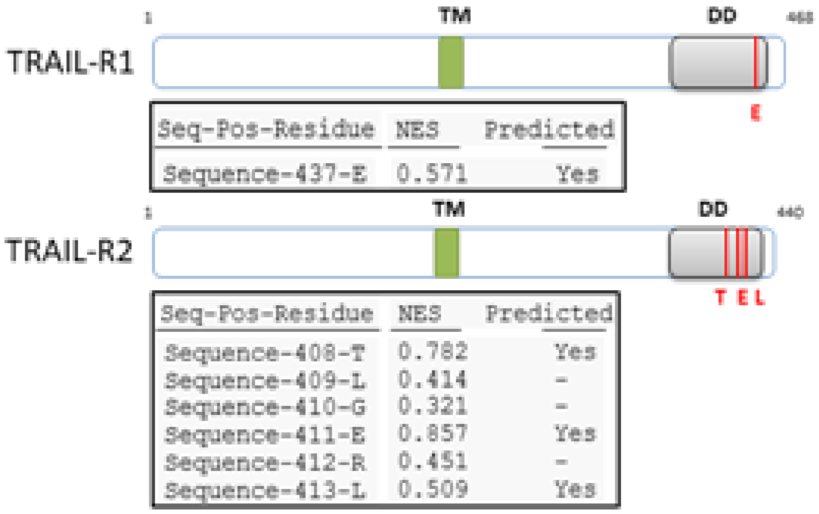

Figure S4. Predicted nuclear export sequence (NES) in TRAIL-R1 and TRAIL-R2 analyzed by NetNES 1.1 prediction software.

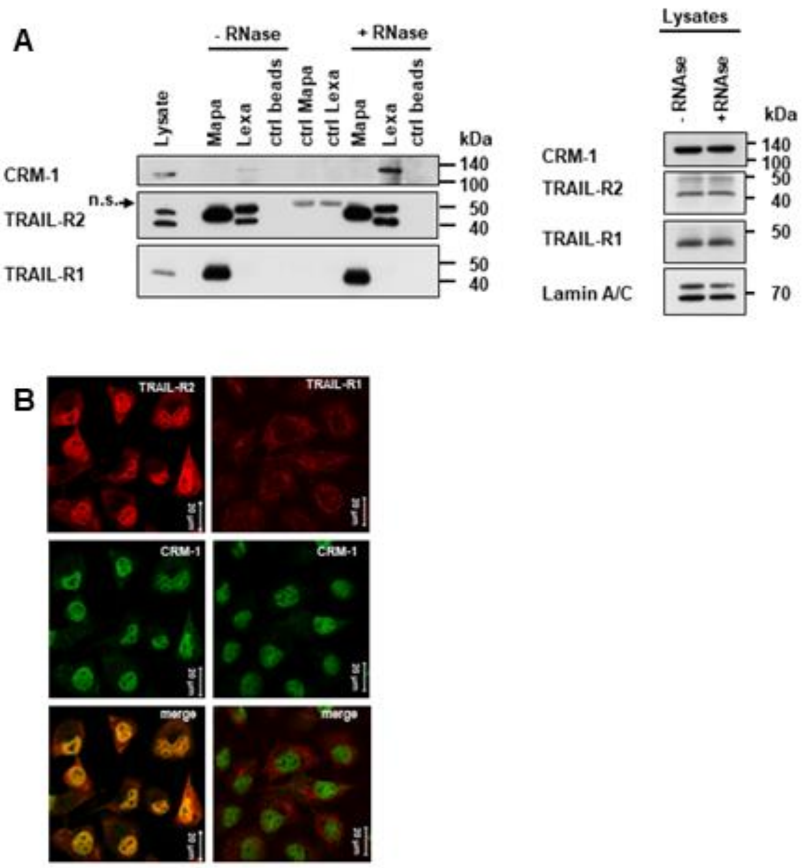

Figure S5. TRAIL-R2 interacts with CRM-1 in Panc-1 cells. (**A**) TRAIL -R1 and -R2 were immunoprecipitated from nuclear fractions of Panc-1 cells using either Mapatumumab (Mapa) or Lexatumumab (Lexa) antibodies. Protein complexes were analyzed by Western blotting. As control,

lysates without antibodies (Ctrl beads) and antibodies alone (CTRL Mapa, CTRL Lexa) were analyzed in parallel. Immunoprecipitation were also performed with nuclear extracts pretreated with RNase. Impact of the RNase-treatment on the levels of studied proteins was analyzed by Western blotting. (**B**) Co-localization of TRAIL-R1 and -R2 with CRM1 was analyzed by indirect immunofluorescence and confocal LSM in Panc-1 cells.

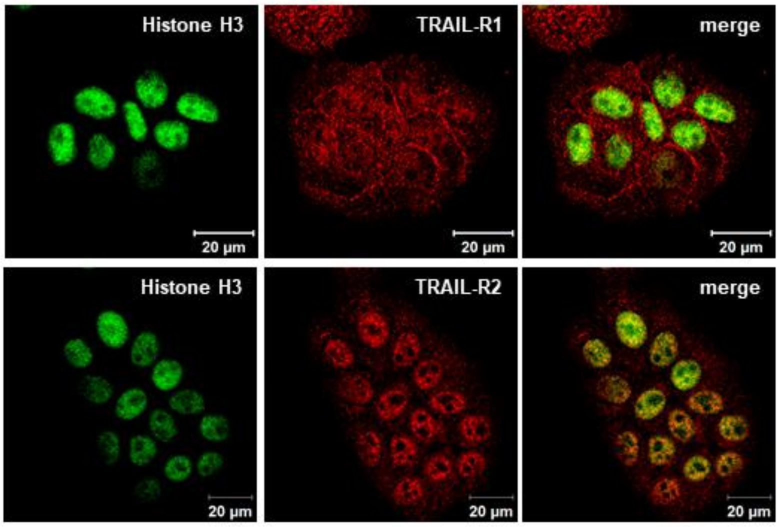

Figure S6. Co-localization of TRAIL-R1 or TRAIL-R2 with Histone H3 in Panc89 cells studied by immunofluorescence and confocal LSM.

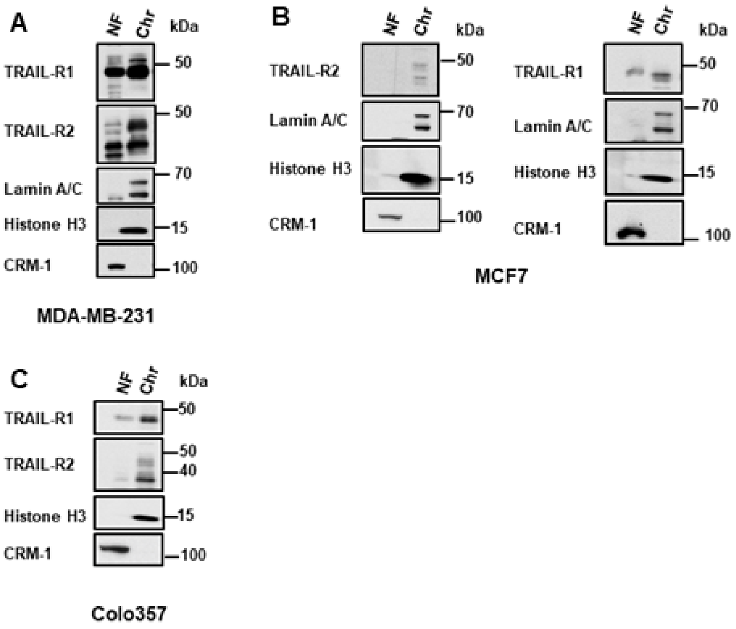

Figure S7. TRAIL death receptors localized in the chromatin. Chromatin fractions (Chr) and supernatants (SN) from breast cancer cell lines (MDA-MB-231 and MCF7) and pancreatic cancer cell line (Colo357) were analyzed by immunoblotting using the indicated antibody. Subcellular fractionation was done as mentioned previously [79].

© 2019 by the authors. Licensee MDPI, Basel, Switzerland. This article is an open access article distributed under the terms and conditions of the Creative Commons Attribution (CC BY) license (http://creativecommons.org/licenses/by/4.0/).
